# Assembly of continuous high‐resolution draft genome sequence of *Hemicentrotus pulcherrimus* using long‐read sequencing

**DOI:** 10.1111/dgd.12924

**Published:** 2024-04-17

**Authors:** Tetsushi Komoto, Kazuho Ikeo, Shunsuke Yaguchi, Takashi Yamamoto, Naoaki Sakamoto, Akinori Awazu

**Affiliations:** ^1^ Graduate School of Integrated Sciences for Life Hiroshima University Higashi‐Hiroshima Japan; ^2^ Department of Genomics and Evolutionary Biology National Institute of Genetics Shizuoka Japan; ^3^ Shimoda Marine Research Center University of Tsukuba Shimoda Japan; ^4^ Research Center for the Mathematics on Chromatin Live Dynamics Hiroshima University Higashi‐Hiroshima Japan

**Keywords:** ArsInsC, *cis*‐regulatory sequences, long‐read sequencing, sea urchin genome, repeat sequences

## Abstract

The update of the draft genome assembly of sea urchin, *Hemicentrotus pulcherrimus*, which is widely studied in East Asia as a model organism of early development, was performed using Oxford nanopore long‐read sequencing. The updated assembly provided ~600‐Mb genome sequences divided into 2,163 contigs with N50 = 516 kb. BUSCO completeness score and transcriptome model mapping ratio (TMMR) of the present assembly were obtained as 96.5% and 77.8%, respectively. These results were more continuous with higher resolution than those by the previous version of *H. pulcherrimus* draft genome, HpulGenome_v1, where the number of scaffolds = 16,251 with a total of ~100 Mb, N50 = 143 kb, BUSCO completeness score = 86.1%, and TMMR = 55.4%. The obtained genome contained 36,055 gene models that were consistent with those in other echinoderms. Additionally, two tandem repeat sequences of early histone gene locus containing 47 copies and 34 copies of all histone genes, and 185 of the homologous sequences of the interspecifically conserved region of the *Ars* insulator, ArsInsC, were obtained. These results provide further advance for genome‐wide research of development, gene regulation, and intranuclear structural dynamics of multicellular organisms using *H. pulcherrimus*.

## Graphical abstract

Assembly of the continuous high‐resolution draft genome sequence of sea urchin, *Hemicentrotus pulcherrimus*, was performed using long‐read sequencing. Two long tandem repeats of early histone genes loci, various *cis*‐regulatory repeat sequences, and 185 of the homologous sequences of *Ars*‐insulator core region were identified. These results provide further advances for genome‐wide research of development, gene regulation, and intranuclear structural dynamics of multicellular organisms using *H. pulcherrimus*.

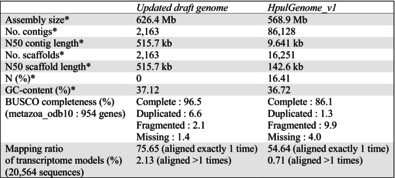

## 1 INTRODUCTION

Sea urchins are model organisms commonly used for studying early development and morphogenesis due to their evolutionary position as diverged at the early period of deuterostome evolution. For example, *Hemicentrotus pulcherrimus*, which is one of the typical sea urchins in East Asia, has been widely investigated to reveal developmental systems of multicellular organisms such as intranuclear chromatin structural dynamics, establishment of left–right asymmetric body axis, and nervous system formation (Matsushita et al., 2017; Takemoto et al., 2016; Yaguchi & Katow, 2003). The gene regulatory network controlling endomesoderm specification in sea urchin embryos was also extensively studied using *Strongylocentrotus purpuratus* (Davidson et al., 2002; Oliveri & Davidson, 2004).

The detailed features of genome sequences of various organisms promote research for a variety of universal and specific activities of entire living systems. Therefore, similar to various organisms, sea urchins draft genomes have also been assembled; for example, *S. purputarus* in 2006 (Sodergren et al., 2006) (the latest version was updated in 2019), *H. pulcherrimus* in 2018 (Kinjo et al., 2018), *Lytechinus variegatus* in 2020 (Davidson et al., 2020), *Temnopleurus reevesii* in 2022 (Kinjo et al., 2022), and *Paracentrotus lividus* in 2023 (Marletaz et al., 2023).

Recent research using the Hi‐C method and its derivatives (Lieberman‐Aiden et al., 2009), which has progressed rapidly in the last decade, has revealed that the expression of each gene is affected by various *cis*‐regulatory elements and their higher‐ordered structures such as enhancer–promoter loops, nucleosome exclusive nonlooping insulator sequences (NENLIS) (Matsushima et al., 2019), topologically associated domains (TADs), A/B compartments, etc. (Lieberman‐Aiden et al., 2009). To analyze the effects of such *cis*‐regulatory structures at various scales ranging from several kb to Mb, a highly contiguous genomic sequence longer than several Mb of the target organism is required.

However, it is difficult to construct such a long contiguous scaffold that contains a transcribed gene region and its *cis*‐regulatory elements such as enhancers and insulators with conventional short‐read‐based genome assembly. For example, the previously reported draft genome sequence of *H. pulcherrimus*, called HpulGenome_v1, did not contain the *Ars* insulator sequence located 2 kb upstream of the arylsulfatase (*Hp‐Ars*) gene (Akasaka et al., 1999; Takagi et al., 2012), which has an interspecies‐conserved AT‐rich core region (ArsInsC) known as a typical NENLIS (Matsushima et al., 2019). Furthermore, the *Hp‐Ars* gene‐containing scaffold in the HpulGenome_v1 did not include another important regulatory region, the C15 enhancer (Iuchi et al., 1995; Sakamoto et al., 1997). Since repetitive sequences are dispersed throughout the genome, the presence of repeat sequences leads to difficulty in the short‐read‐based genome assembly. In addition, it was impossible to reconstruct long repeat sequences far exceeding several 100 bp, which frequently appear in multicellular organism genomes; therefore, many gaps had to exist in the scaffold. Thus, although it has been suggested that the *H. pulcherrimus* genome contains long repetitive sequences consisting of 10–100 of tandem sequences of genes encoding histones H1, H2A, H2B, H3, and H4, HpulGenome_v1 does not contain such long repeat regions.

However, the above problems are being overcome by technological advances in long‐read sequencing, which yields sequences of 10–100 kb per read. At present, reads obtained by long‐read sequencing appear to contain errors 100 times more frequently than those obtained by short‐read sequencing (Logsdon et al., 2020; Rang et al., 2018). However, the hybrid assembly method, which assembles short reads from the same organism while correcting errors in long reads, may enable highly accurate genome assembly with long continuity by taking advantage of both (Holley et al., 2021).

Therefore, in this study, the *H. pulcherrimus* draft genome sequence was updated by hybrid assembly using both long reads obtained using the Oxford Nanopore sequencer and short reads used in conventional *H. pulcherrimus* draft genome assembly. In this paper, we compared the updated draft genome sequence with HpulGenome_v1, and provided the annotation of genes by comparison with related species. We also show that the updated draft genome contains the ArsInsC sequence at upstream of the *Hp‐Ars* gene and its homologous sequences in the updated genome. Furthermore, we identified two distinct long tandem repeats of early histone genes. Such improvements of the draft genome sequence will drive further progress of studies for physiological and structural–dynamical features of gene regulatory behaviors in *H. pulcherrimus*.

## 2 MATERIALS AND METHODS

### 2.1 Sample collection and DNA extraction

Adult males of *H. pulcherrimus* were collected from the sea around Etajima City, Hiroshima Prefecture, Japan, with permission of the Hiroshima Fishery Cooperative, and the genomic DNA (gDNA) was extracted from the sperm of three adult individuals. The *H. pulcherrimus* genomic DNA was extracted by using the Blood & Cell Culture DNA Midi Kit (QIAGEN), and short genomic DNA segments were removed by the Short Read Elimination XL Kit (Circulomics Inc.). To obtain extremely long reads, we followed the protocol described by Logsdon et al. (2020).

### 2.2 Sequencing library preparation

Nanopore sequencing libraries were individually prepared from gDNA derived from each sample using the Rapid Sequencing Kit (SQK‐RAD004; Oxford Nanopore Technologies [ONT]). Nanopore sequencing were performed on an ONT MinION sequencer using R9.4.1 flow cells and FAST5 files were basecalled to FASTQ files using ONT MinIT (MNT‐001). In the following, each of the generated FASTQ formatted reads was named ONT‐read.

### 2.3 Preprocessing of illumina short reads and ONT read data

The preprocessing (adapter trimming and removal of low‐quality reads) of Illumina short reads from gDNA of *H. pulcherrimus* (DRR107784, DRR107785, and DRR107786) and transcriptome (DRR107783) were performed using fastp v0.22.0 (Chen et al., 2018). Error correction of ONT read data derived from each library was individually performed using Ratatosk v0.7.0 (Holley et al., 2021) with preprocessed Illumina short reads (160.47 Gb in total).

### 2.4 Genome assembly and polishing

De novo genome assembly for *H. pulcherrimus* was performed with over 5 kb error corrected and merged ONT reads using Raven v1.8.1 (Vaser & Šikić, 2021), Flye v2.8.3‐b1695 (Kolmogorov et al., 2019), and Wtdbg2 v2.5 (Ruan & Li, 2020). These draft genome sequences were corrected with each other and a single consensus sequence was generated using MAECI (Lang, 2022). This consensus sequence was polished with Illumina short reads using MAECI.

### 2.5 Assessment of draft genome sequences

To evaluate the completeness of the genome assembly, highly conserved genes in metazoan were searched using BUSCO v5.3 (Manni et al., 2021). As an additional analysis, predicted transcriptome models (nonredundant open reading frames [ORFs] derived from transcriptome assembly in HpBase, HpulTranscriptome_nucl.fa) were mapped to each draft genome sequence using hisat2 v2.2.1 (Kim et al., 2019).

### 2.6 Gene prediction and gene annotation

The preprocessed Illumina short reads from the transcriptome were mapped to draft genome sequences using hisat2 v2.2.1 (Kim et al., 2019), and BAM formatted files were generated using SAMtools v1.17 (Li et al., 2009). Gene prediction was performed using these BAM files by BRAKER2 v2.1.4 (Brůna et al., 2021). Gene annotation (orthology search) of gene models predicted by BRAKER2 was performed using BLASTP v2.6.0 (Altschul et al., 1990) with protein models in HpBase and Echinobase (*Strongylocentrotus purpuratus* v5.0 and *Lytechinus variegatus* v3.0). In addition, homologous gene models were searched using BLASTP v2.6.0 with SwissProt and NCBI nr database.

## 3 RESULTS

### 3.1 Genome assembly and completeness of draft genome sequence

Sequencing by the ONT MinION sequencer generated 17.29 million reads with 51.54 Gb of sequenced data. By the error correction and filtering out ONT reads, 2.76 million reads with 35.26 Gb were generated for genome assembly. The draft genome sequence was updated by assembling and polishing these reads by Raven, Flye, Wtdbg2, and MAECI (Table 1, Table S1). Here, the contig (contig ID: Utg196084) that was most homologous to the mitochondrial genome of *H. pulcherrimus* [NC_023771.1] by BLASTN was removed. The updated draft genome sequence consists of 2,163 contigs with a total length of ~626.4 Mb and N50 length of ~515.7 kb (Table S1), with a smaller number of contigs and more continuously assembled than HpulGenome_v1; HpulGenome_v1 consists of ~16,000 scaffolds (contigs) with total length of ~600 Mb containing a total of ~100 Mb gaps and N50 = 142.6 kb (Table S1).

**TABLE 1:** Summary of genome assembly, BUSCO completeness and gene annotation in HpulGenome_v1 and our draft genome sequence.

Version of genome assembly	Updated draft genome	HpulGenome_v1
Assembly size^a^	626.4 Mb	568.9 Mb
No. contigs^a^	2,163	86,128
N50 contig length^a^	515.7 kb	9.641 kb
No. scaffolds^a^	2,163	16,251
N50 scaffold length^a^	515.7 kb	142.6 kb
*N* (%)^a^	0	16.41
GC‐content (%)^a^	37.12	36.72
BUSCO completeness (%) (metazoa_odb10: 954 genes)	Complete: 96.5	Complete: 86.1
	Duplicated: 6.6	Duplicated: 1.3
	Fragmented: 2.1	Fragmented: 9.9
	Missing: 1.4	Missing: 4.0
Mapping ratio of transcriptome models (%) (20,564 sequences)	75.65 (aligned exactly 1 time)	54.64 (aligned exactly 1 time)
	2.13 (aligned >1 times)	0.71 (aligned >1 times)

^a^ Calculated from BBtools (Bushnell et al., 2017) (stats.sh).

Analysis of genome completeness of the updated draft genome by BUSCO using the metazoa_odb10 showed a larger value of 96.5% (89.9% single copy and 6.6 duplicated) than that of HpulGenome_v1 (86.1% [84.8% single copy and 1.3 duplicated]). Additionally, the mapping ratio of transcriptome models of the present draft genome also obtained a larger value of 77.78% than that of HpulGenome_v1 (55.35%).

### 3.2 Gene prediction based on genome assembly and gene annotation with related species

The analysis by BRAKER2 and transcriptome data (DRR107783) predicted 46,914 genes exist in the updated draft genome sequence. By eliminating the predicted genes that were too short (less than 50 amino acids), contained multiple stop codons, or were encoded on the mitochondrial contig, 46,826 gene models were obtained in the updated draft genome, with 5.85 exons per gene model and an average coding sequence (CDS) length of 1,249.23 bp (Table S2). The inference from the orthology relationships between the present gene models and gene models in other sea urchins through reciprocal BLASTP searches showed that 36,055 gene models (number of reciprocal best hit pair, 20,434 and number of not reciprocal but best hit, 15,621) were homologous (best hit with *e*‐value ≤ 1e−10) to publicly known gene models of *H. pulcherrimus*, *S. purpuratus*, or *L. variegatus* (Tables 2 and S2).

**TABLE 2:** Summary of BLASTP searches with protein models of HpBase and other sea urchin.

Subject	Reciprocal best hit pair	Not reciprocal but best hit	No hit
*H. pulcherrimus*	15,652	16,847	14,327
(HpBase, HpulGenome_v1_prot.fa)	(33.43%)	(35.98%)	(30.60%)
*S. purpuratus*	16,153	17,190	13,483
(Echinobase, sp5_0_GCF_proteins.fa)	(34.50%)	(36.71%)	(28.79%)
*L. variegatus*	14,802	16,613	15,411
(Echinobase, Lvar3_0_GCF_proteins.fa)	(31.61%)	(35.48%)	(32.91%)
*P. lividus* *	14,958	18,388	13,480
	(31.94%)	(39.27%)	(28.70%)
*H. pulcherrimus*, *S. purpuratus*, or *L. variegatus*	20,434	15,621	10,771
	(43.64%)	(33.36%)	(23.00%)

* Proteins fasta file of *Paracentrotus lividus* was created using gffread v0.12.7 (Pertea & Pertea, 2020). The genome and annotation in GTF format of *P. lividus* were downloaded from Zenodo (https://zenodo.org/record/7459274).

### 3.3 Detection of early histone gene loci with long tandem repeats

The *H. pulcherrimus* genome has been suggested to contain two nonallelic early histone gene loci that are the long repetitive sequences consisting of 10–100 of tandem sequences of genes encoding histones (Table S3) (Matsushita et al., 2017). However, such long tandem repeat sequences could not be detected by recent short‐read‐based genome assemblies; in HpulGenome_v1, BLASTN searches did not find any genomic regions homologous even to single repeat sequences of early histone loci.

In contrast, two contigs containing the regions of long tandem repeats of histone genes were found on the updated draft genome sequence; where one contig (contig ID: Utg198178, with 479,333 bp) involved 47 tandem repeats, and the other contig (contig ID: Utg200276, with 127,611 bp) involved 34 tandem repeats (Table S3).

### 3.4 Regulatory sequences of arylsulfatase gene

We have investigated the regulatory mechanism of the transcription of the arylsulfatas*e* (*Hp‐Ars*) gene. The promoter (−252 to +38) is the minimum region required for temporal expression (Iuchi et al., 1995; Morokuma et al., 1997). The C15 enhancer required for large enhancement of the expression is present in the first intron (Iuchi et al., 1995; Sakamoto et al., 1997). The polypyrimidine sequence (−2,201 to −1,680) that can form an intramolecular triplex structure (Sakamoto et al., 1996; Yamamoto et al., 1994) and the *Ars* insulator (−2,686 to −2,109) (Akasaka et al., 1999) are present in the upstream region.

In HpulGenome_v1, scaffold989 contains the the *Hp‐Ars* gene. A BLASTN search revealed that this scaffold includes all exons, the promoter, and upstream sequences to the polypyrimidine region. However, it does not include the C15 enhancer in the first intron and the *Ars* insulator in the upstream flanking region, which play important roles in the transcriptional regulation.

**FIGURE 1:**
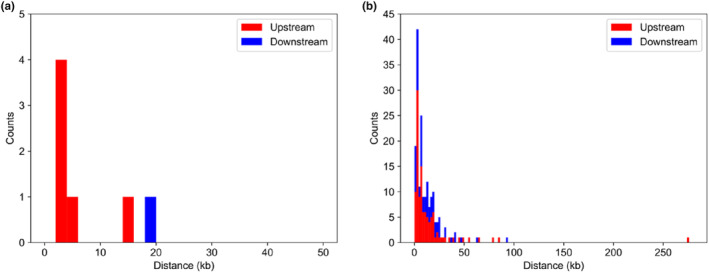
Stacked histogram of genomic distances between DIR1 homologs and their nearest gene (a), and that between DIR2 homologs and their nearest gene (b). The height of red and blue bars indicates counts of direct repeats (DIR) homologs at the upstream and downstream region of the nearest gene, respectively. Distances between DIR homolog and its nearest gene was defined as the genomic distance between the centers of them.

On the other hand, in the updated draft genome, the *Hp‐Ars* gene‐containing contig (contig ID: Utg200732) contains all exons and regulatory sequences including *Ars* insulator and C15 enhancer. Furthermore, the *Hp‐Ars* gene has been reported to include various types of repetitive sequences such as two direct repeats (DIR, which are called DIR1and DIR2, respectively) and an inverted repeat (INV) in the upstream region (Akasaka et al., 1994). All of these repetitive sequences could be found in contig Utg200732. Interestingly, when we searched homologous sequences of the inverted repeat (−475 to −217) with 90–120 bp of the unit length, >90% identity and >90% query coverage among inverted repeats were obtained from IRF v3.08 (Warburton et al., 2004), and nine homologous inverted repeat sequences were found in the updated draft genome (Table S4). Although seven out of these homologous inverted repeats were present in intergenic regions, there was no tendency in their position. Furthermore, eight homologous sequences of the direct repeat DIR1 (−3,440 to −3,109) with two repeat units, >90% identity, and >95% query coverage were found, and 205 homologous sequences of another direct repeat DIR2 (−3,096 to −2,592) with two to five repeat units, >90% identity, and >95% query coverage were found in the updated draft genome (Table S5). These DIR1 and DIR2 homologs tend to exist in the upstream region of the nearest genes (Figure 1).

### 3.5 Homologous genome region to *Ars* insulator

**FIGURE 2:**
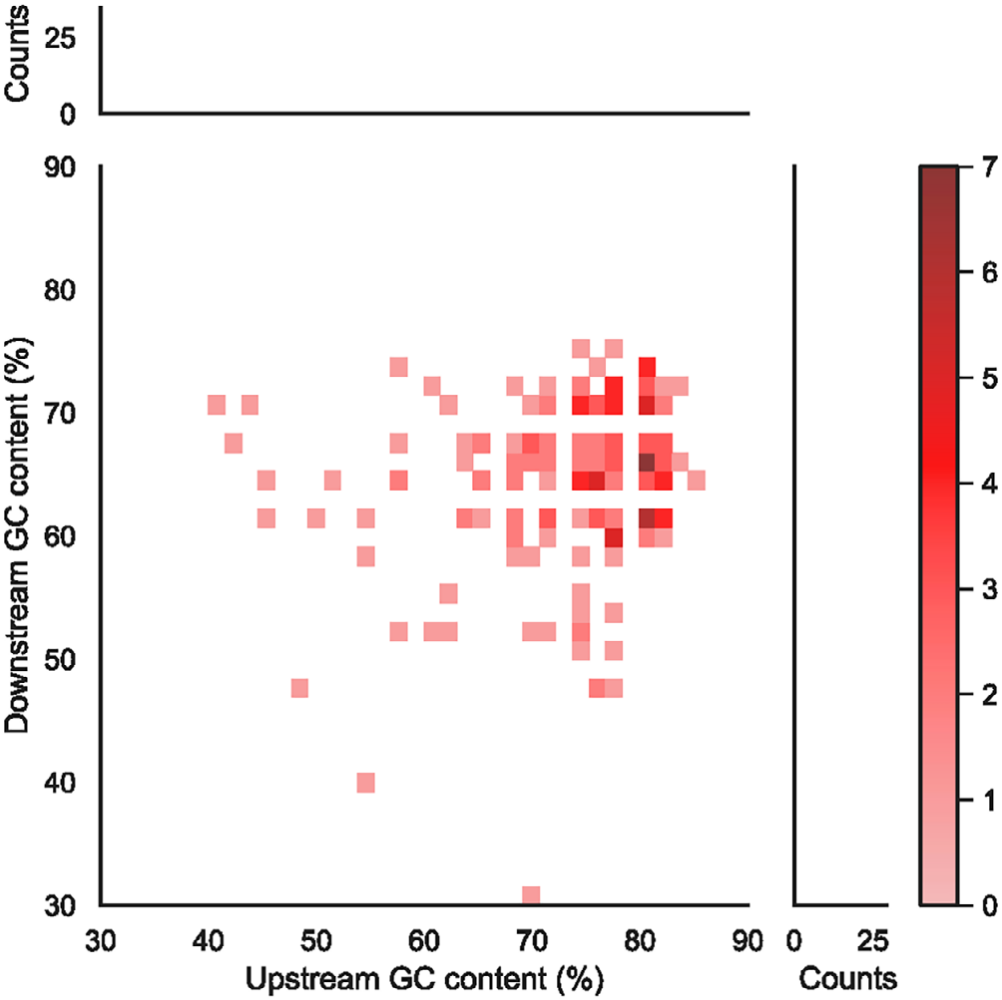
Heat map of the number of ArsInsC homologs with each GC content in the upstream 50 bp and downstream 50 bp regions. The histograms represent the projection onto the upstream GC content axis and the downstream GC content axis, respectively.

The *Hp‐Ars* gene has been known to involve the *Ars* insulator, which includes the interspecifically conserved ArsInsC sequence (Akasaka et al., 1999; Takagi et al., 2012) (Table S4) that is a typical example of NENLIS (Matsushima et al., 2019). In HpulGenome_v1, the *Hp‐Ars* gene was present, but the ArsInsC sequence was not present. In contrast, the updated draft genome contained ArsInsC from 46,417 to 46,236 (minus strand) in contig ID: Utg200732, which is 2‐kb upstream of the *Hp‐Ars* gene region. ArsInsC was originally identified by interspecific sequence comparison with 75% identity in the 30 bp of the sliding window and does not have specific motifs except its interspecies‐conserved AT‐rich sequence (Takagi et al., 2012). Therefore, to find candidate sequences for functional homologs of ArsInsC, nucleotide sequences that have more than 90% identity to ArsInsC were searched. BLASTN analysis found 185 sequences that have more than 90% identity to ArsInsC (Table S6). As observed in the *Ars* insulator, the 50‐bp regions surrounding the ArsInsC homologous sequences tended to have high GC contents (50%–80% GC) (Figures 2 and S1), and 121 homologous sequences (65.4%) contained a G(C)‐stretch of more than 8 bp in these GC‐rich regions (Figure S1). Thus, the sequence properties of the *Ars* insulator are well conserved in these homologous sequences, suggesting that these homologous sequences may function as insulators in the sea urchin genome.

### 3.6 Short tandem repeat

It has been reported that short tandem repeats (STRs) are enriched in gene promoters and variable repeat number is associated with gene expression (Sawaya et al., 2013; Vinces et al., 2009). Furthermore, approximately 90% of transcription factors preferentially bind to STRs (Horton et al., 2023). Therefore, we also analyzed the presence of STRs in the updated draft genome using TRF v4.09 (Benson, 1999). According to the definition of microsatellite by Sawaya et al. (2013), uninterrupted consecutively repeated units of one to four nucleotides that are at least 12 bp in length were searched. After searching STRs that obtained from TRF results and matched this definition, a total of 111,553 mononucleotide repeats, 66,665 dinucleotide repeats, 61,983 trinucleotide repeats, and 62,448 tetranucleotide repeats were found in the updated draft genome (Table S7). However, the enrichment of STRs in the proximity of genes or the translation start sites was not observed.

### 3.7 Transposable elements prediction

Transposable elements (TEs) were predicted and classified using RepeatModeler v2.0.5 (Smit, AFA, Hubley, R. *RepeatModeler Open‐1.0*. 2008–2015, http://www.repeatmasker.org) and RepeatMasker v4.1.5 (Smit, AFA, Hubley, R & Green, P. *RepeatMasker Open‐4.0*. 2013–2015, http://www.repeatmasker.org) for the updated draft genome of *H. pulcherrimus*, and the genomes of *S. purpuratus*, *L. variegatus*, and *P. lividus*, respectively. Most TEs were not annotated as known elements, but the classification of TEs and their proportions indicated that the trends of TEs in the updated draft genome sequence were closer to those in other sea urchins than in HpulGenome_v1 (Table S8).

### 3.8 Microsynteny plots

Microsyntenies were plotted for the five fragment sets of *H. pulcherrimus* contig and *S. purpuratus* scaffold in order of having the most reciprocal BLASTP best hit pairs (Figure S2). While genome‐wide synteny plots showed little interchromosomal gene order rearrangement (data not shown), microsynteny plots showed intrachromosomal gene order rearrangement, similar to the results of genome‐scale synteny plots in other sea urchins (Marletaz et al., 2023).

## 4 DISCUSSION

Long‐read sequencing and hybrid assembly using long and short reads were performed to update the *H. pulcherrimus* draft genome with more continuous and higher accuracy than the recently reported HpulGenome_v1. This study resulted in a draft genome of *H. pulcherrimus* with higher values of the metrics by BUSCO and higher mapping rate of the transcriptome model than HpulGenome_v1.

This draft genome, involving 46,826 gene models, is larger than HpulGenome_v1 containing 24,860 model genes. Here, 36,055 of these gene models were annotated based on the analysis with the homologous gene models of related sea urchins.

The present draft genome contained two long repeat sequences, including the tandemly connected early histone genes. This result may drive the progress of genomic and epigenomic analyses to reveal the developmental stage‐dependent changes in intranuclear location and interactions of early histone loci (Matsushita et al., 2017).

Additionally, this draft genome also contained ArsInsC sequence and more than 100 ArsInsC homologous sequences. Furthermore, the number of ArsInsC homologous sequences with more than 75% identity in the updated draft genome was 241. In vertebrates, the CTCF‐binding sequence is known as a typical insulator sequence responsible for gene expression control. Recently, however, it has been suggested that sea urchin CTCF functions during mitosis rather than interphase (Watanabe et al., 2023). Therefore, ArsInsC and its homologous sequences may play an important role in the control mechanism of sea urchin interphase gene expression, and the present draft genome may promote research to clarify the mechanism.

The updated draft genome involved more than 10,000 nonannotated genes. These genes were expected to include noncoding RNA‐derived or *H. pulcherrimus*‐specific genes. The annotation of these gene models should be progressed by the comparison among genes in various organisms and the function analysis by experiments in the future. Additionally, a large number of duplicated genes were found by analysis with BUSCO in the present draft genome compared with HpulGenome_v1. The reason for this was not clear, nor whether each duplication is true in *H. pulcherrimus*. Further validation should be a future research direction.

Sawaya et al. (2013) suggested that enrichment of STRs in promoters by calculating distances from transcription start site to STR. However, since gene model estimation based on mapping results of bulk RNA‐sequencing did not show the transcription start site and the distribution of the 5′‐UTR length in the *H. pulcherrimus* genome, we analyzed the distribution of distances from translation start sites to STRs. Although the enrichment of STRs in the proximity of genes and translation start site was not observed in this study, it is necessary to examine the enrichment of STRs in gene promoters after the estimation of transcription start site from other sequencing methods such as CAGE‐sequencing or Iso‐sequencing.

In this study, HpulGenome_v1 was updated using Oxford Nanopore long‐read sequencing, resulting in a smaller number of contigs with more continuous assembly. Since each contig was expected to contain a complete set of *cis*‐regulatory sequences, we believe that this updated draft genome improves the applicability of *H. pulcherrimus* genome information to various genome‐wide analyses.

HpulGenome_v1 was assembled using genomic DNA isolated from *H. pulcherrmius* collected in Shimoda, Shizuoka, Japan (Kinjo et al., 2018). However, the sea urchin genome is highly polymorphic (Britten et al., 1978; Sodergren et al., 2006; Yamamoto et al., 2007). For genome‐wide analysis of *H. pulcherrimus*, a genomic sequence that can be widely used is required. Therefore, to perform genome‐wide analysis reflecting the degree of polymorphism in *H. pulcherrimus*, we used genomic DNA isolated from multiple individuals of *H. pulcherrimus* collected in Hiroshima. After error correction and genome assembly, there were 1,625 mixed bases on the updated draft genome sequence (Table S9), and the draft genome size was restrained to ~600 Mb, although the estimated genome size is ~800 Mb (Kinjo et al., 2018). We expect that excessive redundancy is suppressed, and the updated draft genome represents polymorphism in *H. pulcherrimus*. On the other hand, genes determined to be duplicated in BUSCO and the contigs containing them are likely to be redundant. However, we did not remove these contigs because they may contain important information such as polymorphic regions when editing the genome of *H. pulcherrimus*. In addition, it is difficult to perform quantitative haplotype‐resolved analysis based on *k*‐mer distribution with raw reads obtained from the Oxford Nanopore sequencer because of the high error rate of sequencing by this sequencer. Thus, we plan to correct the redundancy of draft genome sequences by, for example, using the Hi‐C method, in the future.

## Supporting information


**Table S1.** Final updated draft genome sequence (FASTA format) (a), and features of respectively obtained assembly results by Raven, Flye, and Wtdbg2 (b).
**Table S2.** Gene models and their features obtained by present assembled draft genome. Nucleotide and amino acid sequences of each gene model (FASTA format) (a) and (b), gene transfer format (GTF) description of each gene position and feature (c), and gene annotation (d).
**Table S3.** Sequence of early histone locus (a), and locations of long repeated histone genes in HpulGenome_v1 and updated draft genome (b–d). Raw results obtained by BLASTN search for HpulGenome_v1 (b) and updated draft genome (c), and locations of units of repeated histone genes (d).
**Table S4.** Sequence of Ars‐INV (a) and subsequence used for BLASTN search (FASTA format) (b), and locations of homologous sequences to Ars‐INV in updated draft genome (c). Raw results obtained by BLASTN search (d).
**Table S5.** Subsequences of DIR1 (a) and DIR2 (b) (FASTA format), and location of homologous sequences to DIR1 (c) and DIR2 (d) in updated draft genome. Raw results obtained by BLASTN search of DIR1 (e) and DIR2 (f ).
**Table S6.** Sequence of ArsInsC (a), locations of homologous sequences to ArsInsC in updated draft genome (b). Raw result obtained by BLASTN (c).
**Table S7.** Locations of STRs in updated draft genome.
**Table S8.** Percentage of transposable elements in updated draft genome (a), HpulGenome_v1 (b), *Strongylocentrotus purpuratus* (c), *Lytechinus variegatus* (d), and *Paracentrotus lividus* (e).
**Table S9.** Counts of mixed‐base in update draft genome.
**Figure S1.** Upstream (left) and downstream (right) 50 bp sequences of 185 ArsInsC homologs and their locations. Guanine (G) and cytosine (C) are colored in red and orange, respectively. The region indicated by black arrowhead contains G(C)‐stretch.
**Figure S2.** Microsynteny plots for the five fragment sets of *Hemicentrotus pulcherrimus* contig and *Strongylocentrotus purpuratus* scaffold in order of having the most reciprocal BLASTP best hit pairs, Utg196122 (6559–2,129,850) and NW_022145600.1 (32,990,742–36,732,968) (a), Utg196144 (2815–3,067,557) and NW_022145596.1 (14,003,427–19,491,491) (b), Utg196236 (14,595–3,147,059) and NW_022145597.1 (16,612,579–30,528,068) (c), Utg196730 (2,747,835–65,719) and NW_022145612.1 (8,056,827–11,267,206) (d), and Utg197110 (40,911–2,168,173) and NW_022145612.1 (4,796,676–9,222,589) (e).

## Data Availability

The sequence data of the updated genome assembly were featured on HpBase (https://cell-innovation.nig.ac.jp/Hpul/) with the name HpulGenome_kure_v1. The raw sequencing data of whole genome sequencing was deposited in the DDBJ Sequence Read Archive (DRA) with accession DRA017089, and was also submitted to DDBJ/EMBL/GenBank databases (BioProject accession PRJDB16611).
