# Supplementary material for: Assembly of continuous high‐resolution draft genome sequence of Hemicentrotus pulcherrimus using long‐read sequencing
Source: Dev Growth Differ. 2024 Apr 17;66(4):297–304. doi: 10.1111/dgd.12924 (PMC11457506; doi:10.1111/dgd.12924)
Supplement: Supplementary file 1 — Table S1. Final updated draft genome sequence (FASTA format) (a), and features of respectively obtained assembly results by Raven, Flye, and Wtdbg2 (b). Table S2. Gene models and their features obtained by present assembled draft genome. Nucleotide and amino acid sequences of each gene model (FASTA format) (a) and (b), gene transfer format (GTF) description of each gene position and feature (c), and gene annotation (d). Table S3. Sequence of early histone locus (a), and locations of long repeated histone genes in HpulGenome_v1 and updated draft genome (b–d). Raw results obtained by BLASTN search for HpulGenome_v1 (b) and updated draft genome (c), and locations of units of repeated histone genes (d). Table S4. Sequence of Ars‐INV (a) and subsequence used for BLASTN search (FASTA format) (b), and locations of homologous sequences to Ars‐INV in updated draft genome (c). Raw results obtained by BLASTN search (d). Table S5. Subsequences of DIR1 (a) and DIR2 (b) (FASTA format), and location of homologous sequences to DIR1 (c) and DIR2 (d) in updated draft genome. Raw results obtained by BLASTN search of DIR1 (e) and DIR2 (f ). Table S6. Sequence of ArsInsC (a), locations of homologous sequences to ArsInsC in updated draft genome (b). Raw result obtained by BLASTN (c). Table S7. Locations of STRs in updated draft genome. Table S8. Percentage of transposable elements in updated draft genome (a), HpulGenome_v1 (b), Strongylocentrotus purpuratus (c), Lytechinus variegatus (d), and Paracentrotus lividus (e). Table S9. Counts of mixed‐base in update draft genome. Figure S1. Upstream (left) and downstream (right) 50 bp sequences of 185 ArsInsC homologs and their locations. Guanine (G) and cytosine (C) are colored in red and orange, respectively. The region indicated by black arrowhead contains G(C)‐stretch. Figure S2. Microsynteny plots for the five fragment sets of Hemicentrotus pulcherrimus contig and Strongylocentrotus purpuratus scaffold in order of having the most reciproca [file DGD-66-297-s001.zip › RE_HP_ReAssembly_sup.docx]

Supplementary information

Table S1: Final updated draft genome sequence (FASTA format) (a), and features of respectively obtained assembly results by Raven, Flye, and Wtdbg2 with error-corrected ONT-reads, and Raven with raw ONT-reads (b). There is a remarkable difference in the mapping rate of transcriptome models between draft genome sequences generated by Raven using raw reads and that using error-corrected reads.

(a) “HpulGenome_kure_v1_contig.fa” in “https://cell-innovation.nig.ac.jp/cgi-bin/Hpul_public/Hpul_annot_download.cgi”

(b)

|  | Raven | Flye | Wtdbg2 | Raven (with raw ONT-reads) |
| --- | --- | --- | --- | --- |
| Assembly size* | 619.2 Mb | 985.5 Mb | 851.0 Mb | 629.2 Mb |
| No. contigs* | 2,164 | 22,260 | 16,967 | 2,208 |
| N50 contig length* | 508.4 kb | 144.8 kb | 240.3 kb | 550.5 kb |
| No. scaffolds* | 2,164 | 22,175 | 16,967 | 2,208 |
| N50 scaffold length* | 508.4 kb | 146.1 kb | 240.3 kb | 550.5 kb |
| N (%)* | 0 | 0 | 0 | 0 |
| GC-content (%)* | 36.91 | 37.36 | 36.94 | 36.71 |
| BUSCO completeness (%)  (metazoan_odb10:  954 genes) | Complete : 96.1  Duplicated : 6.5  Fragmented : 1.8  Missing : 1.8 | Complete : 96.6  Duplicated : 37.5  Fragmented : 2.6  Missing : 0.8 | Complete : 90.3  Duplicated : 10.4  Fragmented : 3.8  Missing : 5.9 | Complete : 91.8  Duplicated : 2.5  Fragmented : 4.2  Missing : 4.0 |
| Mapping ratio of  transcriptome models (%)  (20,564 sequences) | 71.93 (aligned exactly 1 time)  3.12 (aligned >1 times) | 69.48 (aligned exactly 1 time)  8.00 (aligned >1 times) | 59.26 (aligned exactly 1 time)  1.85 (aligned >1 times) | 57.45 (aligned exactly 1 time)  0.35 (aligned >1 times) |

Table S2: Gene models and their features obtained by present assembled draft genome.

Nucleotide and amino acid sequences of each gene model (FASTA format) (a) and (b), gene transfer format (GTF) description of each gene position and feature (c), and gene annotation (d).

(a) “HpulGenome_kure_v1_nucl.fa” in “https://cell-innovation.nig.ac.jp/cgi-bin/Hpul_public/Hpul_annot_download.cgi”

(b) “HpulGenome_kure_v1_prot.fa” in “https://cell-innovation.nig.ac.jp/cgi-bin/Hpul_public/Hpul_annot_download.cgi”

(c) “https…/HpulGenome_kure_v1.gtf” in “https://cell-innovation.nig.ac.jp/cgi-bin/Hpul_public/Hpul_annot_download.cgi”

(d) “HpulGenome_kure_v1.1_annot_revised.xlsx” in “https://cell-innovation.nig.ac.jp/cgi-bin/Hpul_public/Hpul_annot_download.cgi”

Table S3: Sequence of early histone locus (a), and locations of long repeated histone genes in HpulGenome_v1 and updated draft genome (b-d). Raw results obtained by BLASTN search for HpulGenome_v1(b) and updated draft genome (c), and locations of units of repeated histone genes (d).

(a) “HpEarlyHistone.fa”

(b) “HpulGenome_v1_BLASTN_withEarlyHistoneLoci.csv”

(c) “HpulGenome_kure_v1_BLASTN_withEarlyHistoneLoci.csv”

(d) “HpulGenome_kure_v1_EarlyHistoneLoci_47_34copies.csv”

Table S4: Sequence of *Ars*-INV (a) and subsequence used for BLASTN search (FASTA format) (b), and locations of homologous sequences to *Ars*-INV in updated draft genome (c). Raw results obtained by BLASTN search (d).

(a) “HpArs-INV.fa”

(b) “HpArs-INV_left.fa”

(c) “HpulGenome_kure_v1_HpArs-INV_homologue.csv”

(d) “HpulGenome_kure_v1_BLASTN_withHpArs-INV.csv”

Table S5: Subsequences of DIR1 (a) and DIR2 (b) (FASTA format), and location of homologous sequences to DIR1 (c) and DIR2 (d) in updated draft genome. Raw results obtained by BLASTN search of DIR1 (e) and DIR2 (f).

1. “HpArs-DIR1.fa”
2. “HpArs-DIR2.fa”
3. “HpulGenome_kure_v1_HpArs-DIR1_homologue.csv”

(d) “HpulGenome_kure_v1_HpArs-DIR2_homologue.csv”

(e) “HpulGenome_kure_v1_BLASTN_withHpArs-DIR1.csv”

(f) “HpulGenome_kure_v1_BLASTN_withHpArs-DIR2.csv”

Table S6: Sequence of ArsInsC (a), locations of homologous sequences to ArsInsC in updated draft genome (b). Raw result obtained by BLASTN (c).

(a) “HpArsInsC.fa”

(b) “HpulGenome_kure_v1_ArsInsC_homologue.csv”

(c) “HpulGenome_kure_v1_BLASTN_withArsInsC.csv”

Table S7: Locations of STRs in updated draft genome.

“HpulGenome_kure_v1_ShortTandemRepeats.csv”

Table S8: Percentage of transposable elements in updated draft genome (a), HpulGenome_v1 (b), *S. purpuratus* (c), *L. variegatus* (d), and *P. lividus* (e).

“TransposableElements.xlsx”

Table S9: Counts of mixed-base in update draft genome.

“Mixed-base_CountsTable.xlsx”


 Fig. S1: Upstream (left) and downstream (right) 50 bp sequences of 185 ArsInsC homologs and their locations. Guanine (G) and cytosine (C) are colored in red and orange, respectively. The region indicated by black arrowhead contains G(C)-stretch.

Fig. S2: Microsynteny plots for the five fragment sets of *H. pulcherrimus* contig and *S. purpuratus* scaffold in order of having the most reciprocal BLASTP best hit pairs, Utg196122 (6,559 to 2,129,850) and NW_022145600.1 (32,990,742 to 36,732,968) (a), Utg196144 (2,815 to 3,067,557) and NW_022145596.1 (14,003,427 to 19,491,491) (b), Utg196236 (14,595 to 3,147,059) and NW_022145597.1 (16,612,579 to 30,528,068) (c), Utg196730 (2,747,835 to 65,719) and NW_022145612.1 (8,056,827 to 11,267,206) (d), and Utg197110 (40,911 to 2,168,173) and NW_022145612.1 (4,796,676 to 9,222,589) (e). The horizontal arrows represent the position and orientation of orthologous genes. Orthologs are represented in the same color, and curves are drawn between their translation start sites.
